# Weighing up the pros and cons of dysphagia triage in South Africa

**DOI:** 10.4102/sajcd.v70i1.941

**Published:** 2023-02-21

**Authors:** Kelly-Ann Kater, Jaishika Seedat

**Affiliations:** 1 Department of Speech Pathology, School of Human and Community Development, Faculty of Humanities, University of the Witwatersrand, Johannesburg, South Africa

**Keywords:** dysphagia, triage, emergency unit, South Africa, speech–language therapy, evidence-based practice; dysphagia triage

## Abstract

**Background:**

Early identification of dysphagia followed by intervention reduces, length of hospitalisation, degree of morbidity, hospital costs and risk of aspiration pneumonia. The emergency department offers an opportune space for triage. Triaging offers risk-based evaluation and early identification of dysphagia risk. A dysphagia triage protocol is not available in South Africa (SA). The current study aimed to address this gap.

**Objectives:**

To establish the reliability and validity of a researcher-developed dysphagia triage checklist.

**Method:**

A quantitative design was used. Sixteen doctors were recruited from a medical emergency unit at a public sector hospital in SA using non-probability sampling. Non-parametric statistics and correlation coefficients were used to determine the reliability, sensitivity and specificity of the checklist.

**Results:**

Poor reliability, high sensitivity and poor specificity of the developed dysphagia triage checklist was found. Importantly, the checklist was adequate in identifying patients as not being at risk for dysphagia. Completion time for dysphagia triage was 3 minutes.

**Conclusion:**

The checklist was highly sensitive but not reliable or valid for use in identifying patients at risk for dysphagia.

**Contribution:**

The study provides a platform for further research and modification of the newly developed triage checklist, which is not recommended for use in its current form. The merits of dysphagia triage cannot be ignored. Once a valid and reliable tool is confirmed, the feasibility of implementation of dysphagia triage must be considered. Evidence to confirm that dysphagia triage can be conducted, when considering the contextual, economic, technical and logistic aspects of the context, is necessary.

## Background

Dysphagia, that is, failed or impaired bolus transit, may be caused by either a mechanical or inflammatory response (Triggs & Pandolfino, 2019). To mitigate aspiration, aspiration pneumonia, morbidity and mortality, length of hospitalisation and associated medical costs, early detection of dysphagia by way of screening is advocated (Sherman et al., 2021). For screening to have the desired outcome, that is, a pass or fail indication of dysphagia, adherence to parameters is compulsory. Additionally, regular and consistent implementation will positively impact the ability or practice of screening and identification of dysphagia (George et al., 2009). Sherman and colleagues confirmed that the dysphagia screening rate for patients with stroke in the United States and China ranged from 56.7% to 80.8% (Sherman et al., 2021). This variability in screening suggests that not every patient is being screened, and the risk of patients with dysphagia being missed still exists even in developed contexts.

Reasons underpinning the variable implementation of standardised screening tools in well-resourced contexts are important to consider when understanding how best to accommodate screening in less-resourced and challenging contexts. The South African public healthcare sector may well be considered such a context, being fast-paced, inadequately staffed, suboptimally resourced and patient-heavy (Brooke-Sumner et al., 2019). Khoza-Shangase (2021) highlighted the challenges facing our South African public healthcare context and despite her content talking to challenges around implementation of Universal New-born Hearing Screening, the challenges she identified hold true when conceptualising a dysphagia screening undertaking. Thus, a viable alternative, as advocated by Pierpoint and Pillay (2020), is the use of *informal* screening practices in dysphagia. Informal screening would constitute a focused observation of clinical signs and symptoms without a formal checklist or tool which generally has specified pass or fail criteria (Pierpoint & Pillay, 2020). An alternative that alleviates the increased demands associated even with a screening (which is in any case quicker than a diagnostic assessment but which nevertheless still requires staff training, use of foods and liquids, as well as time) is triage. Prioritising patients according to medical need and urgency before their entry into the hospital emergency room safeguards the usage of available personnel, equipment and beds, which may be in short supply. This underpins triage and its value in a resource-constrained context (Wuerz et al., 2000). Constraints intrinsic to the public health sector context in South Africa and which have been supported by Pillay and Pillay (2020) include imbalanced speech therapist to patient ratios, patient discharge prior to consultation by the speech therapist, high workloads of speech therapists and an inability by speech therapists to see all the patients who require their services, hence poor service delivery. Globally, screening for dysphagia may be undertaken by nurses as well. In a study by Heaton, Farrell and Bassett (2019) in Australia on nurse-led (*n* = 3726) long-term screening over a 9-year period, an average hospital-wide compliance rate of 74% and an accuracy rate of 82% with regard to dysphagia were reported. Importantly, a survey on nurse satisfaction associated with the screening showed high levels of satisfaction. Outcomes from nurse-led screening protocols show positive results and success (Cornwell et al., 2016; Lees et al., 2006). Given the challenges with workload and staffing among nurses in South Africa (Morton et al., 2020), adding screening to their duties is an aspect that will require further research before implementation. Literature abounds with evidence on the association of dysphagia to aspiration pneumonia, malnutrition, weight loss, frequent hospital admission with prolonged length of stay, increased mortality and decreased quality of life (Altman et al., 2010; Carrión et al., 2015). Simply put, mistimed intervention increases the management burden in an already vulnerable patient group with consequent increased economic cost to the healthcare institution and sector (Patel et al., 2018).

The challenge in a resource-constrained context is staff availability to conduct the screening (whether it be by speech therapists, nurses or any health professional), training prior to implementing screening, time availability and willingness to undertake the screening regularly. These variables pose a challenge in the government health sector in South Africa and are likely to curtail implementation of a dysphagia screening protocol in any government health facility, similar to the challenges identified by Khoza-Shangase in terms of newborn hearing screening. It is thus prudent to consider how dysphagia may be identified quickly and early to navigate optimal intervention, and if not by screening, then an alternative way.

Research on dysphagia triage is scarce, with only one research study published in this area (Cichero et al., 2009). More recently, an opinion piece on triage in South Africa did shed critical insights on triage as a means to address early identification of dysphagia for patients who may be at risk (Kater, 2022). The scarcity of research in this area requires critical reflection. One is cautioned against making assumptions about inadequacy around the notion of triage in dysphagia or assumptions about poor feasibility leading to limited publications in this area. The proposed study will try to provide more insight on why triage in dysphagia continues to remain a reasonable proposition that could be viable in challenging and constrained healthcare contexts. Dysphagia triage is a cost-effective method that does not require additional personnel or resources (Broussard & Altschuler, 2000; Cichero et al., 2009). Not requiring training but rather identification based on presenting symptomatology facilitates easy completion of a checklist pertaining to swallowing variables. The use of a dysphagia triage checklist would allow for immediate patient identification, timely referral for ongoing care and reduced waiting time. The triage concept enables one to identify the presence or absence of dysphagia within a short space of time without the use of food or liquids and informs the need or not for further diagnostic assessment by a speech therapist (Cichero et al., 2009; Jean, 1990). Implications for patient prognosis are central to triage, with positive outcomes enhanced for variables such as complications, finances, length of hospital stay and so forth.

Dysphagia triage for all patients who come into the hospital emergency department will provide an opportune time and space to identify if that patient is at risk for dysphagia, regardless of the reason for admission. Thus, the outcome of triage would be identification of risk for dysphagia and necessary intervention thereafter (Cichero et al., 2009). Given the key role of doctors in accident and emergency departments, knowledge about dysphagia is valuable. Cichero et al. (2009), in their study, successfully integrated nurses and doctors into the critical role of swallow triage. The rationale behind the study by Cichero et al. (2009) was to reduce the risk of a patient commencing oral intake inappropriately or unsafely, as poor nutritional intake may intensify costs related to recovery and length of stay of the patient. Dysphagia triage would ensure that patients receive the correct diet, thus preventing incorrect diet allocation and food wastage. Additionally, identification and provision of assistance to patients who require assistance when eating may be attributed to dysphagia triage (Cichero et al., 2009). Triage protocols maximise the use of limited available resources and keep patients and providers safe.

### Triage versus screening versus diagnostic assessment for dysphagia in a resource-constrained context such as South Africa

Regardless of the route chosen by a speech therapist to identify dysphagia in a patient, the result is what remains important (Etges et al., 2014), as it ultimately initiates the management of dysphagia. Globally, and as supported by Etges et al. (2014), diagnostic standardised assessment and screening protocols remain widely known to those practising in dysphagia, and they remain heterogenous in nature, with many having been developed for:

- specific patient populations, for example, adults, paediatrics, geriatrics (Denuit et al., 2021)
- specific underlying pathology, for example, stroke, head and neck cancer (Audag et al., 2019)
- implementation by a particular professional, such as the nurse or speech therapist (Oliveira et al., 2019).

**FIGURE 1:**
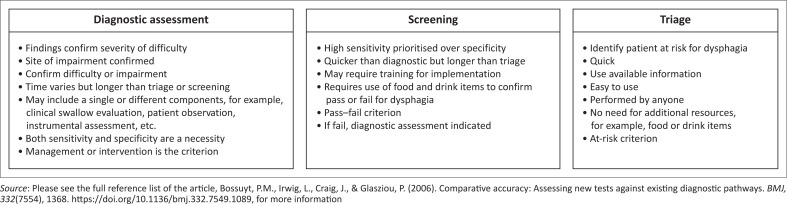
Considerations for clinical-decision making when choosing among diagnostic assessment versus screening versus triage.

The context in which one is practising ultimately dictates choice. Figure 1 elucidates some considerations linked to choice.

Given the features linked to a triage tool, and the evidence from Khoza-Shangase (2021) commenting on the challenges associated with screening, it seems necessary to investigate triage as a method to identify patients with dysphagia or at risk for dysphagia. One is reminded that with the current status of government hospitals in South Africa, that is, poorly resourced, overcrowded, understaffed and underfunded (Maphumulo & Bengu, 2019; Rajan & Englebrecht, 2018), it is uncertain if a dysphagia triage protocol could be accommodated within the current functioning of the accident and emergency unit. The time to complete an additional area of triage by doctors and their willingness to take this on (i.e., staff attitude) are unknown.

The current study was therefore a first step to establish the feasibility of dysphagia triage in public-sector hospitals. The current study included the compilation of a triage tool as well as implementation of the tool. Currently, no dysphagia triage protocol exists for the South African context. While the current study investigated the reliability and validity of a self-developed triage checklist, the authors will critique the relevance of a standardised triage tool and challenge dysphagia therapists to critically reflect on what the end goal pertaining to dysphagia is. Is it a standardised tool that in being compliant loses its relevance and becomes arduous to complete, or is it a quick triage checklist that is more likely than not to alert us to a patient at risk for dysphagia, knowing that these patients were likely to have been missed anyway? Is this not what is wanted and needed for a resource-constrained context?

## Method

The aims of the study were to compile a dysphagia triage checklist following a review of current screening tools and thereafter to establish the reliability and validity of the developed dysphagia triage checklist. A quantitative design was used. Sixteen medical staff participated in the study. The participants were medical staff working at the MEU, and they were selected using purposive convenient sampling. Thus, the researchers did not impose any changes or restrict criteria for inclusion or exclusion. Any medical staff member who was allocated to work at the MEU and who agreed to participate in the study was recruited. This contributed toward the validity of the tool, as participant criteria aligned with the reality of the context.

All patients who were treated at the MEU at the time of data collection were triaged for dysphagia as well. To establish the sensitivity and specificity of the triage checklist, it was necessary for all patients who underwent the dysphagia triage to be screened for dysphagia, regardless of pass or fail. The researchers completed the dysphagia screening but were blinded to the results of the triage until after the screening was completed. The South African Dysphagia Screening Tool (SADS) (Ostrofsky & Seedat, 2016) was used.

**FIGURE 2:**
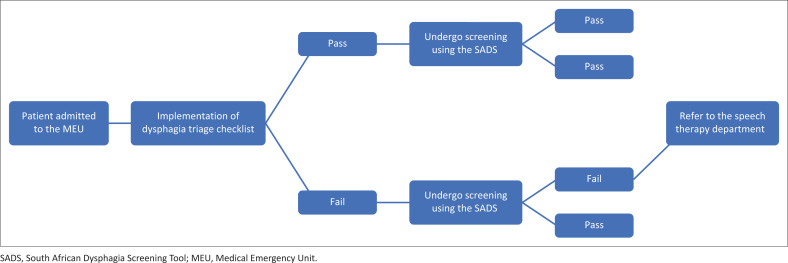
Procedure followed for patients admitted to the Medical Emergency Unit at the hospital.

As per Figure 2, any patient who failed the SADS was referred for diagnostic assessment of dysphagia, regardless of the results of the triage. The diagnostic dysphagia assessments were completed by the Speech Therapy Department at the research site.

The dysphagia triage checklist was developed by the researchers for the study. It was developed following a review of dysphagia screening tools to establish the items for inclusion in the checklist. The following dysphagia screening tools were reviewed and adapted:

- The Standardised Swallow Assessment (Perry, 2001a, 2001b)
- Massey Bedside Swallowing Screening (Massey & Jedlicka, 2002)
- the Yale Swallow Protocol (Leder & Suiter, 2014)
- the SADS (Ostrofsky & Seedat, 2016).

The developed dysphagia triage checklist was divided into four sections and consists of eight test items (Appendix 1). The dysphagia triage checklist was administered by the doctors in the MEU over an 8-week period.

### Description of the dysphagia triage checklist

#### Section A

Items 1, 2 and 3 were aimed at determining the patient’s level of alertness as well as their ability to maintain an adequate respiratory status. A decreased level of alertness and state of consciousness may affect the patient’s ability to swallow safely (Cichero, 2006). Respiratory-compromised patients may experience swallowing difficulty as the effort required to maintain an adequate respiratory rate may make swallowing a challenging and demanding task, thus putting the patient at risk of dysphagia (Cichero, 2006).

#### Section B

Item 4 was aimed at determining whether or not the patient can manage their own saliva. Poor saliva management could be indicative of poor head control, inability to close the mouth, abnormal tongue mobility and reduced intra-oral sensation (Cichero, 2006). Additionally, a gurgling vocal quality after swallowing of secretions may indicate pooled material in the pharynx (Cichero, 2006).

Item 5 assessed the patient’s ability to produce voice. The ability to produce voicing provided information regarding laryngeal functioning (Cichero & Murdoch, 2006). If a patient is unable to produce voicing when they receptively understand the instruction, this may be indicative of respiratory problems and laryngeal weakness (Cichero & Murdoch, 2006; Murray, 1999). The true vocal folds may not be fully adducting as a result of paralysis, trauma or disease leading to less or no protection of the airway and aspiration (Cichero, 2006). Additionally, the presence of wet or ‘gurgly’ breath sounds and/or vocal quality may be indicative of pooled secretions in the pharynx (Johnson & Scott, 2006).

#### Section C

Item 6 determined the patient’s receptive language abilities. The ability of the person to communicate may give important information about the person’s abilities to follow instructions and an example of purposeful oral motor skills (Murray, 1999).

Items 7 and 8 assessed the patients’ ability to voluntarily clear their airway and the type of cough a patient presents with, respectively. Measuring a volitional cough is not a predictor of the patients’ cough reflex in the event of laryngeal penetration or aspiration (Murray, 1999). A volitional cough is not a reflexive cough, but the patient’s ability to cough voluntarily needs to be determined, as there is an increased risk for aspiration in patients who have a weakened voluntary cough. The elicitation of a volitional cough helps the clinician to determine whether the patient is capable of organising the motor movements necessary to clear their airway and expel any penetrated or aspirated material (Murray, 1999). A productive cough produces phlegm or mucus (sputum), while a nonproductive cough is dry and does not produce sputum (Ainslie, 2009). Cough is abnormal if it is persistent, painful or productive (Ainslie, 2009).

### Administration of the checklist

For each test item, the administering doctor was required to indicate the result with a tick mark or an ‘x’. A fail or ‘x’ result for any of the test items indicated that the patient was at risk for dysphagia. The checklist was designed to take under 2 minutes to complete.

### Pass or fail criteria

The administrator of the test was required to indicate a pass or fail result for each test item. A fail result for any of the test items was indicative of a patient being at risk for dysphagia. The data obtained from the use of the dysphagia triage checklist and the results of the dysphagia screening were analysed using quantitative measures, specifically correlational coefficients

### Ethical considerations

Ethical approval to conduct this study was obtained from the University of the Witwatersrand Human Research Ethics Committee (Medical) (ref. no. M160679, 13/09/2017). Medical staff working at the emergency unit were provided with information about the study. After allowing time for them to consider participation, they were required to provide written consent.

## Results

The aims of the study were two-fold: firstly, to compile a dysphagia triage tool, and secondly, to establish the reliability and validity of the tool. As noted above, after critical engagement with several existing tools, a triage tool was compiled and has been described in the methodology above. This section of the results will address the second aim, that is, the reliability and validity of the tool that was developed.

Sixty-seven patients were triaged by doctors at the MEU over an 8-week period.

As can be seen in Table 1, 59.7% (*n* = 40) of the patients passed and 40.2% (*n* = 27) failed the dysphagia triage checklist. As described above, after completion of the dysphagia triage checklist, all 67 patients (regardless of triage result) underwent a dysphagia screening, using the SADS (Ostrofsky & Seedat, 2016) as a measure of the validity of the dysphagia triage checklist. It was vital to establish the validity of the researcher-developed triage checklist to confirm that the checklist was accurate in identifying and prioritising patients in terms of their risk for dysphagia. Results are seen in Table 2.

**TABLE 1:** Results of dysphagia triage (*n* = 67).

Triage results	Frequency	Percentage	Cumulative frequency	Cumulative percentage
Pass	40	59.7	40	59.7
Fail	27	40.3	67	100.0

**TABLE 2:** Results from the dysphagia screening (*n* = 67).

Screening results	Frequency	Percentage	Cumulative frequency	Cumulative percentage
Fail	5	7.46	5	7.46
Pass	62	92. 54	67	100.00

Bearing in mind that 27 patients failed the dysphagia triage, Table 2 highlights that five patients failed the dysphagia screening tool. The results of the SADS indicated that 7.46% (*n* = 5) of the patients presented with dysphagia and 92.54% (*n* = 62) of the patients did not. The percentage of agreement between the dysphagia triage checklist and the dysphagia screening was found to be 59.7%. This means that 59.7% of the patients who were triaged for dysphagia elicited the correct results, that is, correct passes and fails. However, because of the percentage of agreement being criticised as a poor measure of inter-rater reliability, the correlation between the dysphagia triage checklist and the dysphagia screening was worked out using Cohan’s kappa. Cohen’s kappa was calculated as 0.04, which shows poor reliability.

## Reliability: Cohen’s kappa

The relationship between the dysphagia triage checklist and the dysphagia screening assessment (SADS) was evaluated using correlation coefficients (Schiavetti & Metz, 2002). Thus, through the use of Cohen’s kappa, measures of positive agreement and negative agreement provided information regarding the types of agreement that presented between the dysphagia triage checklist and the dysphagia screening assessment (SADS). This is seen in Table 3. Thus, 59.7% of the patients who were triaged for dysphagia elicited the correct results, that is, correct passes and fails. However, Cohen’s kappa is a preferred measure of inter-rater reliability as it incorporates a calculation of hypothetical probability of chance agreements, as opposed to percentage agreement (Wood, 2007).

**TABLE 3:** Calculation of percentage of agreement.

Dysphagia screening results (SADS)	Fail	Pass	Total
Fail	3	3	6
Pass	24	37	61

**Total**	**27**	**40**	**67**

SADS, South African Dysphagia Screening Tool.

The correlation coefficient for Cohen’s kappa can range from -1.0 to +1.0, whereby a kappa of 1.0 is indicative of perfect agreement, and a kappa of 0 shows a poor correlation between the two variables. For the purpose of medical studies and diagnosis, a kappa that lies between 0.40 and 0.70 is an appropriate inter-rater reliability. For this study, Cohen’s kappa is 0.04, which shows poor reliability. Reasons contributing to the poor reliability have been addressed in the discussion.

## Validity

Validity is the degree to which a researcher has measured what they aim to measure (Kumar, 2005). Content validity, face validity and concurrent validity were all included in the research analysis.

### Content validity

Content validity refers to whether the method of measurement measures what it is expected to measure (Schiavetti & Metz, 2002), that is, whether the signs and symptoms of dysphagia are accurately identified by the use of the dysphagia triage checklist. Despite the dysphagia triage checklist being developed after reviewing already-established dysphagia screening tools, it was found not to be specific enough, and as a result of this, patients failed for reasons other than dysphagia, such as being on oxygen. Kaplan and Saccuzzo (2012) explain that there are no statistical measures to determine content validity; thus, such validity is greatly dependent on clinical reasoning, judgement and expertise. It is possible that a higher sample size of patients may have improved the content validity.

### Face validity

Face validity refers to the suitability of a given instrument as a source of data on the subject under investigation (Schiavetti & Metz, 2002). It is based on the users’ judgement, but it is important to consider it as it describes the way the user views the dysphagia triage checklist as valid and has implications for administration of the checklist in a way that is accurate and unbiased. Face validity should have been confirmed through the completion of the questionnaire by doctors in the MEU; however, the response rate was poor. As a result of the poor response rate, face validity cannot be commented on. One must bear in mind the workload and performance demands placed on doctors within the South African context. This study reveals that regardless of minimising the time required to complete the online self-developed questionnaire, even these few minutes were too much.

### Concurrent validity

Concurrent validity is a form of criterion validity. Concurrent validity was important to consider for this particular study. Concurrent validity is concerned with how well a new shorter version of a measure compares to the existing longer measure; that is, how well the dysphagia triage checklist correlates with the dysphagia screening. The percentage of agreement (59.7%) refers to the agreement between the results of the dysphagia triage checklist and the dysphagia screening (SADS).

However, as a result of percentage agreement being a criticised measure, Cohen’s kappa was used and provided information regarding the types of agreement that were present between the dysphagia triage checklist and the SADS. Cohen’s kappa was calculated to be 0.04, which shows poor reliability and thus indicates poor concurrent validity. This means that there is poor correlation between the dysphagia triage checklist and the dysphagia screening.

## Sensitivity and specificity

A measure of sensitivity and specificity of the dysphagia triage checklist was calculated. The measures of sensitivity and specificity are binary classification statistical measures. Sensitivity measures the proportion of true positives which were correctly identified as such (i.e. the percentage of at-risk dysphagia patients who were correctly identified as being at risk of having dysphagia). Specificity measures the proportion of true negatives which are correctly identified (i.e. the percentage of patients with normal swallowing who were correctly identified as not being at risk of dysphagia).

Sensitivity and specificity were calculated, as shown in Table 4. The following calculations were performed:


$$\begin{array}{l} \text{Sensitivity}=(\text{True Positives})/(\text{True Positives}+\text{False Negative})\\ \;=(37)/(3+37)\\ \;=0.92500\;(92.5\%) \end{array}$$ [Eqn 1]

$$\begin{array}{l} \text{Specificity}=\text{True Negatives}/(\text{True Negatives}+\text{False Positives})\\ \;=3/(3+24)\\ \;=0.111111\;(11.11\%) \end{array}$$ [Eqn 2]

**TABLE 4:** Sensitivity and specificity of the dysphagia triage checklist.

Dysphagia screening results (SADS)	Fail	Pass	Total
Fail	3	3	6
Pass	24	37	61

**Total**	**27**	**40**	**67**

*Source:* The South African dysphagia screening tool (SADS): A screening tool for a developing context. *South African Journal of Communication Disorders, 63*(1), Art. #117, 9 pages. https://doi.org/10.4102/sajcd.v63i1.117

SADS, South African Dysphagia Screening Tool.

The dysphagia triage checklist can therefore be defined as being highly sensitive in detecting patients at risk for dysphagia. The specificity of the dysphagia triage checklist is significantly lower than that of the sensitivity; however, it is still adequate in identifying patients as not being at risk for dysphagia when they do not present with the disorder. The specificity calculation indicates that participants failed the dysphagia triage checklist when in fact they did not present with the disorder, based on the results from the screening; that is, false positives were problematic.

## Discussion

The triage checklist that was developed for this study was one that could be completed quickly (within 2 minutes), did not require use of words or written detail but instead the use of a tick or cross and did not require that the healthcare professional completing it be trained. The content of the developed checklist ensured that it could be used for any patient who came into the emergency department; thus, it was not designed for use with a specific diagnosis. No additional resources such as foods or liquids were required, as completion of food trials would be time-consuming and have financial implications. Thus, in addressing the second aim of the current study, the findings confirm that the developed checklist had flaws in that reliability, validity and specificity were poor. Nonetheless, the checklist was sensitive in identifying a patient with or at risk for dysphagia. As a first level of filtering, the potential remains for the checklist investigated in the current study or a modified version of this triage checklist with improved reliability, validity and specificity. In a context where identification of patients with dysphagia is often late, where patients may be discharged before being seen by a speech therapist or where the presence of dysphagia may lead to other complications (Andrews & Pillay, 2017; Stone et al., 2020), the rationale behind the use of dysphagia triage must outweigh the preconceived barriers for reasons explained below.

Initial concerns around the use of triage (for dysphagia) include the pace of work in an emergency department and associated time availability, the priority of lifesaving versus identification of a patient who may be at risk (for dysphagia) and the increased load for (speech) therapists at the hospital, as well as the implication of this should a patient require screening or thereafter diagnostic assessment (Da Costa et al., 2021). It is perhaps because of these very same concerns that triage must be considered as a solution, for while there may be short-term implications, the long-term benefits are likely to outweigh these. Thus, it is valuable to consider the following:

- Any new intervention is initially viewed with trepidation, and there are often forgone conclusions that it will culminate in more work, the need for more staff and perhaps financial implications. Literature confirms, however, that new interventions have long-term multilayered benefits to the institution, professionals, patients and indeed overall service delivery (Wensing & Grol, 2019). There may be an initial phase of familiarisation with the intervention, managing logistics and working through procedures; however, with time and experience, these should resolve, paving the way for the checklist to become part and parcel of the workings of the emergency department.
- Given the rationale and understanding of how triage works and its implications for patients, it is inevitable that there will be an initial increase in patient numbers. However, research confirms that patient numbers do stabilise over a period of time with enhanced throughput (Spencer et al., 2019), given that there should be minimal to no referrals coming via wards or colleagues, except in cases where dysphagia presentation may be late for that patient.
- The goal of early dysphagia identification must remain central. Thus, if triage facilitates identification of patients who may be at risk (based on the signs and symptoms displayed at the emergency department), then this goal is closer to being realised. Patients may not be missed; necessary management may begin, preventing further exacerbation and presentation of complications and comorbidities (Cichero et al., 2009). The likelihood of false positives remains; however, given that the triage takes 2 min and a screening approximately 7 min (Ostrofsky & Seedat, 2016), this ‘wasted time’ is worthwhile.
- For dysphagia therapists working in contexts that are demanding and where delivery of dysphagia services to patients is suboptimal (from a timing perspective), having a realistic perspective on the value of a standardised tool for triage must be queried. The impracticality of this increases as one acknowledges then the requirement for every hospital to have its own tool, given that staffing, structure, functioning and logistics vary across institutions. An easy-to-use, quick and short checklist with minimal requirements from the professional completing it, aside from patient observation, would be ideal. Notwithstanding false positive identification of patients, if those identified at risk are found to have dysphagia, then one must concede that dysphagia intervention is one step closer to the goal of ideal.
- Finally, and an aspect that one may view as being controversial, is that of professional ethics, patients’ rights and *ubuntu* (which is not a single thing but a broad concept involving characteristics such as unselfishness and caring, ‘I am who I am because of you’). In making a choice to work with individuals who are vulnerable, in need of our assistance, advice and services, the implications of the burden this places on the professional cannot be over-emphasised. Being knowledgeable of the consequences of delayed or lack of dysphagia services, speech therapists may not be justified in using contextual or logistical challenges to support their lack of service provision. Recognition that we hold the power to make a difference in a patient’s quality of life must be taken seriously (Iserson, 2020).

The current study shows that triage for dysphagia is possible, and with the correct tool that has reliability and validity, it will provide a more rigorous method to commence early identification and intervention for dysphagia. This will inform considered development of workflow from triage to screening through to diagnostic assessment. With a well-coordinated and documented process from triage through to management, one may have an overview of the value of each step in attaining the overall goal of improved timing of dysphagia intervention, improved patient quality of life and not leaving any patient behind. In the interim, however, the checklist developed in the current study may still provide a preliminary way to filter patients who are and are not at risk for dysphagia and who can be monitored by nursing staff for any swallowing difficulties, if transferred to a ward.

### Limitations

There are some limitations to the current study. The staff at the emergency department did not participate in providing qualitative feedback on the use of the checklist. The addition of qualitative feedback would have provided necessary understanding of the process, implementation of the checklist, barriers, facilitators and recommendations.

Not all emergency departments at the hospital site consented to participate in the study. This limited the number of patients with whom the checklist could be trialled, and the sample of doctors who implemented the checklist was also smaller. Thus, the researchers were not able to gather sufficient depth on the usefulness of the checklist as part of the reliability and validity process.

Gathering data not only from the different emergency units within the same hospital but also from multiple hospital sites would have also provided a broader understanding on the receptiveness of dysphagia triage and provided a larger sample for more detailed analysis.

## Appendix 1

### Dysphagia Triage Checklist

     PLEASE COMPLETE:

Patient Hospital Number: __________________   Patient Name: ___________________________

Name of Hospital: ________________________   Administered by: _________________________

Date of Assessment: ______________________   Date of Screening: ________________________

Medical Diagnosis: _______________________

### INSTRUCTIONS:

- Familiarise yourself with the checklist prior to administering it
- The checklist should take you no longer than 2 minutes to complete
- The checklist should be administered as part of the triage process in the Emergency Department
- For each test item, you will be required to an indicate ‘√ ‘or ‘x’ result.
- The entire checklist must be completed and if there are any ‘x’ a speech therapy referral is indicated
- If you are unsure refer to a Speech Therapist
- Put the original copy in the sealed box for the researcher and the duplicate in the patients file

**Table:**

		indicate ‘√ ‘or ‘x’
	**SECTION A**	
1.	Is the patient awake and alert?	-
2.	Is the patient responsive?	-
3.	Is the patient able to breathe freely? (i.e. no difficulty breathing or maintaining SATS)	-
	**SECTION B**	
4.	Can the patient maintain control of their saliva?	-
5.	Does the patient have a wet or hoarse sounding voice?	-
	**SECTION C**	
6.	Can the patient understand your instructions?	-
7.	Can the patient cough when asked to?	-
8.	Is the patient’s cough productive?	-

Note: Checklist adapted from: The Standardised Swallow Assessment; Massey Bedside Swallowing Screening, Yale Swallow Protocol; the South African dysphagia screening tool (SADS)
